# ERCP after bariatric surgery - literature review and case report

**Published:** 2014-09-25

**Authors:** A Iorgulescu, F Turcu, N Iordache

**Affiliations:** General Surgery Clinic, “Sf. Ioan” Hospital, Bucharest

**Keywords:** ERCP, obesity, bariatric surgery

## Abstract

Abstract

Obesity is a disease which has become more prevalent in Romania. Bariatric surgical procedures are among the treatment options for obese patients. Obesity and the metabolic disorders induced by it are risk factors for gallstones formation and their complications. ERCP is a minimally invasive therapeutic procedure indicated in the treatment of choledochal lithiasis and its complications. ERCP is generally considered the most difficult endoscopic procedure from the technical point of view. The authors have proposed to consider the possibility of performing therapeutic ERCP in patients who have undergone bariatric procedures. Literature data are reviewed and the case of a patient treated in a minimally invasive (laparoendoscopic) way for cholecyst and choledocholithiasis after longitudinal gastrectomy is presented

 The clinical problem 

 Obesity is an important and current health problem, especially in developed countries. In 2012, 68.8% of adults were overweight and 35.7% obese in USA. In these conditions, obesity is no longer just a purely cosmetic issue, but also an important medical condition which is the cause of numerous comorbidities. Therefore, strict measures are necessary to reduce body weight in these patients and, in this context, the bariatric surgery appeared and subsequently developed.

 Pathophysiology and the effects of therapy

 Obesity related metabolic imbalances create the premises for gallstone formation. Obesity causes increased synthesis and secretion of cholesterol, leading to the formation of gallstones. Through its different methods, bariatric surgery leads to a significant and rapid weight decrease. Changes of bile composition, incumbent to body mass reduction, consists of a pronounced increase of mucin concentration (18 times) with an increase of calcium ions (40%). These changes lead to a high propensity of bile stones development.

 A prospective study conducted by Schiffman and published in 1991 in the Am. J. of Gastroenterology showed that in patients undergoing gastric bypass, significant changes of bile composition are generated, which lead to gallstone appearance in 36% of the cases in approximately 6 months and to biliary sludge in another 13% of the cases. Of the patients who developed gallstones, almost half (41%) are symptomatic [**1,2**].

**Fig. 1:**
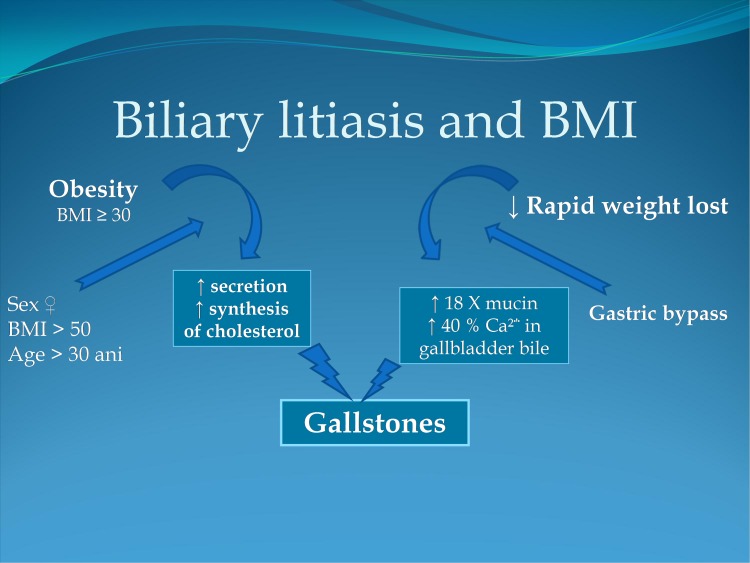
Biliary lithiasis and BMI

 Clinical evidence

 These data have raised new issues, namely the importance of identifying risk factors for the occurrence of gallstones after bariatric interventions. It was found that traditional risk factors for gallstones appearance are not predictive for the formation of biliary lithiasis after bariatric surgery. In 2009, Li published in Surgical Endoscopy the results of a study that identifies the reduction of body mass with at least 25%, as predictive for the formation of gallstones after different bariatric procedures in [**3**].

 Thus, the necessity to develop some strategies for the prevention and treatment of biliary lithiasis and its complications has come into prominence. Some were repeatedly evaluated:

 1. prophylactic administration of ursodeoxycholic acid after bariatric interventions

 2. concurrent laparoscopic cholecystectomy and bariatric surgery in patients without gallstones or with asymptomatic ones.

 3. concurrent laparoscopic cholecystectomy and bariatric surgery in patients with symptomatic gallstones.

 Of these, the currently accepted strategy is to perform laparoscopic cholecystectomy and bariatric surgery concomitantly only for patients with symptomatic gallstones and only if the bariatric intervention is straightforward, otherwise the cholecystectomy will be scheduled for some other time.

 Thus, a significant proportion of patients must be treated for cholelithiasis subsequently. The natural evolution of gallstone in obese patients does not differ from the general population, the spectrum of possible complications being the same.

 The stones migration from the gallbladder to the common bile duct in obese patients who have been subjected to bariatric surgery can lead to complex and severe complications. That is why in these patients who are in a greater perioperative risk, the minimal invasive treatment of common bile duct stones is all the more so important.

 There are various bariatric procedures including restrictive, malabsorbtive and mixed mechanisms to reduce body weight.

**Fig. 2:**
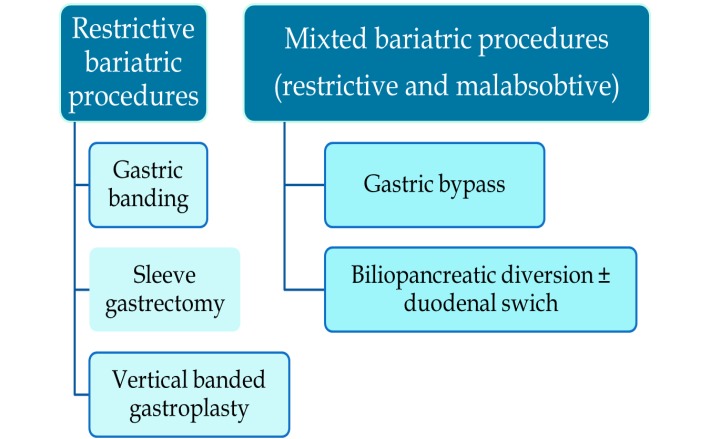
Classification of bariatric procedures

 These procedures cause important anatomical modifications that can be key points with regard to the minimally invasive treatment of chledochal lithiasis. The type of the bariatric procedure is the most important factor in establishing the subsequent therapy, but it is not the only one. Depending upon the type of anastomosis, the approach to the major duodenal papilla can be easy, difficult, or even impossible. The length of the intestinal segments created in some malabsorbtive procedures can affect the endoscopic approach, dictating the need for assisted balloon enteroscopy or echo-guided percutaneous anterograde endoscopy. Besides, the moment that the bariatric procedure took place is of great importance. If the operation is recently done, one can be forced to postpone the endoscopic maneuver until the consolidation of sutures is fulfilled.

 The restrictive bariatric procedures are gastric banding, sleeve gastrectomy and vertical banded gastroplasty.

**Fig. 3:**
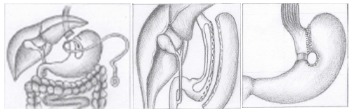
Restrictive bariatric procedures: gastric banding, sleeve gastrectomy and vertical banded gastroplasty

 These enable a transoral route for ERCP. The anatomy is only slightly modified from the perspective of endoscopic papillary access and it allows an easy approach of the biliary ducts.

 Nevertheless, transoral ERCP after bariatric restrictive procedures has some limits:

 1. ERCP has to be delayed until complete healing of the recent staple lines of the anastomosis

2. Deflation of the band in order to accommodate the endoscope, associated or not to endoscopic dilatation 

 The mixed, restrictive – malabsorbtive, bariatric procedures are: gastric bypass and biliopancreatic diversion. The anatomy is significantly modified. After the gastric bypass, the endoscopic access into the biliary tree is difficult, but possible, be it transgastric anterograde laparoscopically assisted or transoral. After the biliopancreatic diversion, the endoscopic access into the biliary tree is impossible because of the length of the interposed intestinal segment.

**Fig. 4:**
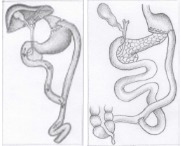
Mixed bariatric procedures: gastric bypass and biliopancreatic diversion

 Each of the different modalities according to which the transoral access into the biliary ducts is achieved has advantages and disadvantages and these are summarized in **Table 1**.

**Table 1:** Transoral access technique after gastric bypass

Transoral access technique after gastric bypass	Advantages	Disadvantages
Lateral view endoscope	• Easy papillary access • Standard accessories	Low rate of success determined by the difficult access into the duodenum
Pediatric colonoscopy or enteroscopy	Deep access	• Lateral view • Lack of elevator • Necessity of special accessories
Simple or double balloon enteroscopy	Deep access	• Lateral view • Lack of elevator • Necessity of special accessories

 In 2011, Lars Aabakken presented, in Munich, the results of a multicentre study including 129 patients with modified anatomy secondary to a bypass procedure with a "Y long loop", 69 of them having a gastric bypass. To these 156 ERCPs have been performed. The techniques used were:

 • ERCP by double balloon enteroscopy 

 • ERCP by simple balloon enteroscopy

 • ERCP by spiral enteroscopy

 Duodenal access had a success rate of 69%, 72% and 74% respectively, with small differences between the three techniques. When duodenal access was accomplished, the success rate of ERCP was of of 88%, with rather similar figures for the different techniques: 63%, 60%, 65%.

 Complications were registered in 12% of cases (16 of 129 patients) and their range was similar to the general complications of ERCP: acute post-ERCP pancreatitis, papillary bleeding or retroperitoneal duodenal perforation [**7**].

 Because transoral access is difficult in patients with gastric bypass, an alternative method was imagined – transgastric access (through the distal stomach). The method implies laparoscopic assistance or the placement of a gastrostomy tube into the distal stomach at the moment of the primary operation (the tube is abandoned in the subcutaneous space, is radiologically traceable and will help a percutaneous access in the distal stomach in the postoperative period). This technique was originally described by Baron [**4**] and Fobi [**5**] in 1998 and recently reiterated by Gutierez [**6**].

 The transgastric access allows the use of an endoscope having lateral view and of standard accessories, the approach to the major papilla being done in the conventional way. The two modalities of transgastric access (laparoscopically assisted and through a gastrostomy tube) have advantages and disadvantages summarized in **Table 2**.

**Table 2:** Transgastric access (distal stomach) of duodenoscope for ERCP

Procedure	Advantages	Disadvantages
Placement of a gastrostomy tube	Allow the repeat procedure with easy access	More invasive than the purely endoscopic technique
Laparoscopically assisted	Also allow an intraperitoneal exploration	Call for a mixed, well coordinated team, surgeon plus endoscopist

 Backup solutions for endoscopic treatment of choledochal lithiasis in patients having mixed bariatric operations (malabsorbtive and restrictive) like gastric bypass and biliopancreatic diversion are:

 • Percutaneous transhepatic anterograde endoscopic access

 • Laparoscopically assisted transenteric endoscopic access

 • Laparoscopic exploration of the common bile duct

 In 2011, Weilert published the results of percutaneous transhepatic endoscopic approach on 8 patients having gastric bypass. The success rate was of 100% and that of choledochal deconstruction was of 67%.

 The endoscopic transenteric access is a difficult approach with just a few case reports in the medical literature [**8,9**] and the laparoscopic exploration of the common bile duct does not have any peculiarities in obese patients with bariatric operations.

 Case report

 We present below the case of a 57-year-old female patient with morbid superobesity and non-symptomatic gallstones.

 The CT examination showed the uncomplicated gallstones and a common bile duct of 7 mm, without images of choledochal lithiasis.

 The patient was scheduled for sleeve gastrectomy and presumed cholecystectomy if allowed (reasonable anesthetic and surgical risk, expeditious and uncomplicated bariatric operation). These conditions being fulfilled, the cholecystectomy was performed. A rather large cystic duct was observed and an intraoperative transcystic cholangiography was done, which detected the presence of multiple choledochal stones.

 Because of the recently performed stapled anastomosis, the endoscopic treatment was delayed under the protection of an external transcystic biliary drainage.

**Fig. 5:**
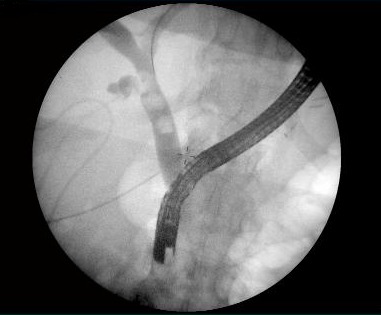
ERCP, multiple choledochal stones

 Two weeks later, a transoral ERCP was done with an easy duodenal access but a papillary one inconveniently influenced by a interposed duodenal diverticula. A sphincterotomy with stones extraction and control cholangiography was performed. The uneventful postoperative course permitted the withdrawal of the external biliary drainage 24 hours later.

## Conclusions

The presence of biliary lithiasis is frequent in obese patients and its natural course is similar to that in the general population. The area of complications is the same and they are not directly linked to the presence of symptoms. Analyzing the therapeutic strategies for the prevention of complication and taking into account the risk/benefit ratio, it was agreed that the conduct to be followed is that which involves the performance of a laparoscopic cholecystectomy with bariatric surgery only for patients with symptomatic lithiasis. 
